# Recent Advances in Transducers for Intravascular Ultrasound (IVUS) Imaging

**DOI:** 10.3390/s21103540

**Published:** 2021-05-19

**Authors:** Chang Peng, Huaiyu Wu, Seungsoo Kim, Xuming Dai, Xiaoning Jiang

**Affiliations:** 1 Department of Mechanical and Aerospace Engineering, North Carolina State University, Raleigh, NC 27695, USA; cpeng6@ncsu.edu (C.P.); hwu15@ncsu.edu (H.W.); 2 Infraredx, Inc., Bedford, MA 01730, USA; skim@infraredx.com; 3 Department of Cardiology, New York-Presbyterian Queens Hospital, Flushing, NY 11355, USA; xud9002@nyp.org

**Keywords:** atherosclerosis, intravascular ultrasound (IVUS) imaging, ultrasound transducer, multifrequency ultrasound imaging, multimodality IVUS imaging

## Abstract

As a well-known medical imaging methodology, intravascular ultrasound (IVUS) imaging plays a critical role in diagnosis, treatment guidance and post-treatment assessment of coronary artery diseases. By cannulating a miniature ultrasound transducer mounted catheter into an artery, the vessel lumen opening, vessel wall morphology and other associated blood and vessel properties can be precisely assessed in IVUS imaging. Ultrasound transducer, as the key component of an IVUS system, is critical in determining the IVUS imaging performance. In recent years, a wide range of achievements in ultrasound transducers have been reported for IVUS imaging applications. Herein, a comprehensive review is given on recent advances in ultrasound transducers for IVUS imaging. Firstly, a fundamental understanding of IVUS imaging principle, evaluation parameters and IVUS catheter are summarized. Secondly, three different types of ultrasound transducers (piezoelectric ultrasound transducer, piezoelectric micromachined ultrasound transducer and capacitive micromachined ultrasound transducer) for IVUS imaging are presented. Particularly, the recent advances in piezoelectric ultrasound transducer for IVUS imaging are extensively examined according to their different working mechanisms, configurations and materials adopted. Thirdly, IVUS-based multimodality intravascular imaging of atherosclerotic plaque is discussed. Finally, summary and perspectives on the future studies are highlighted for IVUS imaging applications.

## 1. Introduction

Cardiovascular disease (CVD) is a collection of diseases and conditions that affect the heart and blood vessels in the heart and other vital organs, including coronary artery disease (CAD), heart failure, stroke and hypertension [1]. According to the data provided by World Health Organization (WHO), CVDs are the No.1 cause of death globally. In 2016, 17.9 million people died from CVDs, which accounts for 31% of all global death; of these deaths, 85% are due to CAD and stroke. It is expected that this number will rise to 23.6 million by 2030 [2].

Atherosclerosis is an inflammatory disorder characterized by the gradual accumulation of lipid-rich plaque in the arterial wall [3,4]. It has been recognized that atherosclerosis is the dominant common cause of CVDs, such as CAD, myocardial infarction and stroke [5]. Even though the exact mechanism of atherosclerotic plaque development has not been elucidated yet [6,7], it is found that the plaques are formed following the lipid accumulation [8]. After some time, the plaques gradually harden and narrow the arterial lumen, thus restricting the blood flow. During this period, the artery may respond to the plaque progression by dilatation of the arterial wall (i.e., arterial positive remodeling). While the positive remodeling may widen the lumen, continuous growth of the plaque will induce the development of neovessels, leading to the instability of the plaques and finally resulting in the plaque rupture [9]. It has been shown that the rupture of atherosclerotic plaques is the major source of acute coronary syndrome (ACS), a common complication of CAD that describes a group of conditions that suddenly stop or severely reduce blood from flowing to the heart muscles [9]. That means atherosclerosis becomes dangerous when plaques rupture and intraluminal thrombus is formed, limiting or blocking the blood flow [10]. Plaques with a high risk of rupture are usually referred to as vulnerable plaques. A vulnerable plaque is featured with a large lipid-rich necrotic core (lipid pool) that is surrounded by a thin fibrous cap and permeated with intraplaque macrophages (i.e., thin-cap fibroatheroma (TCFA)) [11,12]. Studies have shown that the risk of plaque rupture is inversely related with the thin cap thickness; 95% of plaque rupture occurs with a thin cap thickness of <65 µm [13,14]. Therefore, TCFA is viewed as the precursor lesion of plaque rupture; early assessment of TCFA can help in developing therapeutic and interventional strategies to avoid plaque rupture and manage CAD [15,16].

Nowadays, the gold standard for the diagnosis of atherosclerosis is invasive coronary angiography [17]. In this imaging method, a catheter is applied to inject radiopaque liquid contrast agent into the bloodstream first; the blood vessel abnormalities, such as narrowing and blockage, can then be visible on X-ray image [18]. To evaluate a coronary angiogram image, it is necessary to compare the region of narrowing with adjacent normal vessel segments. However, there may be a consequent underestimation of the degree of stenosis if the reference region is diffusely narrowed [19]. Even though invasive coronary angiography shows the advantages, such as locating luminal irregularities with high spatial resolution (~100 µm), and real-time assessment, coronary angiography is a 2D luminogram that cannot provide information about the vessel wall and plaque characteristics [20].

Over the past two decades, various invasive and non-invasive imaging modalities have been introduced and validated to comprehensively identify the structure and composition of atherosclerotic plaques [21]. The invasive imaging modalities include intravascular ultrasound (IVUS) imaging, optical coherence tomography (OCT), near-infrared spectroscopic (NIRS) imaging, intravascular photoacoustic (IVPA) imaging, near infrared fluorescence (NIRF) imaging, time resolved fluorescence spectroscopic (TRFS) imaging and fluorescence life-time imaging (FLIM). The non-invasive imaging modalities include computed tomographic coronary angiography (CTCA), magnetic resonance imaging (MRI) and positron emission tomography (PET). Compared to non-invasive imaging modalities, invasive imaging modalities demonstrate higher spatial and temporal resolutions, which allows for better visualization of luminal stenosis and plaque characteristics. Moreover, most of invasive imaging modalities can provide a real-time assessment of the coronary arteries, whereas non-invasive imaging modalities such as CTCA and MRI require relatively long time of imaging processing [19]. Therefore, the invasive imaging modalities are more popular for assessing the severity of CAD and guiding treatment strategies.

Even though several other invasive intravascular imaging modalities are now available, IVUS imaging so far is the most favorable imaging modality for coronary artery assessment [22,23]. Compared with other intravascular imaging modalities, IVUS imaging provides sufficient spatial resolution and penetration depth for precisely visualization coronary artery anomalies. Moreover, the IVUS images provide real-time cross-sectional view of the arterial wall, including morphological and pathological characteristics [24,25]. In addition to identifying plaque vulnerability features, IVUS imaging has long been validated in clinical without any known short-term and long-term side effects [26].

As one of the most crucial components of an IVUS system, ultrasound transducers produce and detect high-frequency ultrasonic waves for real-time high-resolution imaging. In this review, we aim to extensively examine the recent advances in ultrasound transducers for IVUS imaging applications. Firstly, a fundamental review of IVUS imaging principle, evaluation parameters and IVUS catheters are presented. Then, different types of ultrasound transducers for IVUS imaging are summarized. Particularly, the recent advances in piezoelectric ultrasound transducer for IVUS imaging are extensively examined. Thirdly, IVUS-based multimodality intravascular imaging of atherosclerotic plaque is reviewed. Finally, a summary is presented with perspectives on the future directions for IVUS imaging applications.

## 2. Intravascular Ultrasound (IVUS) Imaging

### 2.1. IVUS Imaging Principle

**Figure 1:**
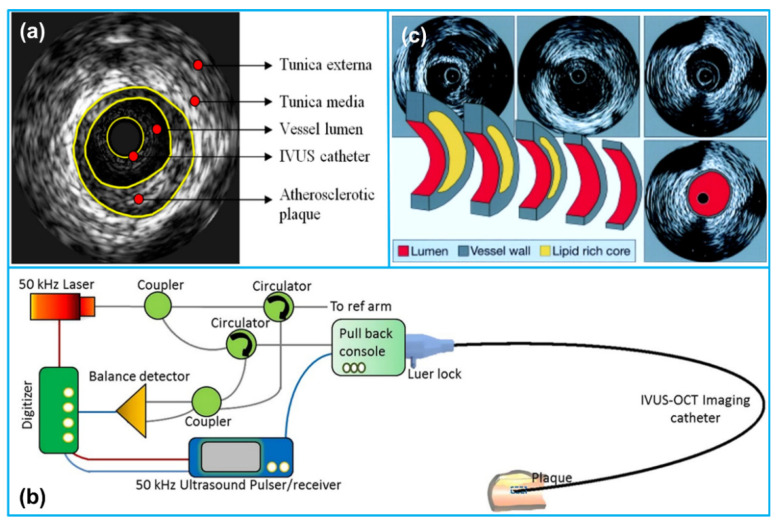
(**a**) A typical grayscale display of an arterial cross-sectional image acquired through intravascular ultrasound (IVUS) imaging. Reprinted from [32] with permission. (**b**) An illustration of an IVUS imaging system composition. Reprinted from [33] with permission. (**c**) Series of tomographic IVUS images acquired through the ultrasound transducer pullback. Reprinted from [30] with permission.

IVUS imaging is an interventional imaging modality that utilizes reflected ultrasound signals to generate high-resolution images of vascular structures. Similar to many other forms of ultrasound imaging, IVUS images are obtained based on the varying acoustic impedance at the interface of different tissue structures. Specifically, the healthy coronary artery wall mainly consists of three layers, including the inner tunica intima, the muscular tunica media and the outer tunica adventitia (Figure 1a) [27]. Atherosclerotic plaque is formed in the inner tunica intima, which has less echogenicity than the highly echo-reflective outer tunica adventitia [28]. However, thin cap fibroatheromas (TCFA) possessing the majority of atherosclerotic lesions demonstrate moderate echogenicity. Since the tunica media that mainly consists of smooth muscle cells does not reflect ultrasound waves, it appears dark in the grayscale ultrasound image, permitting easy identification of the three layers.

An IVUS imaging system usually consists of three major components: a catheter incorporating a small ultrasound transducer or an array, an imaging console including the necessary hardware and software to convert the acquired IVUS signals to images, and a pullback device to withdraw the catheter in the models with auto-pullback system (Figure 1b) [29]. During IVUS imaging, an IVUS catheter with a tiny transducer at its distal end is advanced over a guidewire beyond the target lesion. Once the catheter is in place, the ultrasound transducer is electrically activated and high frequency ultrasound waves, typically centered at 20–60 MHz, are emitted and propagate into different tissues [30]. A pullback of the transducer tip through the lesion or segment of interest is then performed at a speed of 0.5–2.0 mm/s using the automatic pullback device [31]. The continuously acquired reflection signals from the arterial wall will be utilized to generate a series of tomographic images of the vessel wall (Figure 1c). For example, using a typical pullback speed of 0.5 mm/s and an imaging frame rate of 30 images/s, 60 grayscale images will be obtained from a pullback through a 1 mm segment. Compared with auto-pullback, manual pullback is currently more commonly used, which provides the operator with the opportunity to focus on specific areas of vascular wall more carefully.

Even though grayscale IVUS imaging enables real-time, high-resolution tomographic assessment of atherosclerosis in vivo, delineating plaque area and distribution as well as lesion length based on the comparison of the echogenicity of the plaque to its surrounding adventitia [34], it is limited with regard to quantitative assessment of the plaque composition within a lesion. For instance, both dense fibrotic and calcified tissues in plaques have strong echo-reflections and are thus difficult to differentiate based on grayscale IVUS imaging. In recent two decades, various mathematical methods have been developed to post-process the radiofrequency (RF) backscatter signal, which can generate sophisticated images of atherosclerotic tissue composition. Clinically, there are three different kinds of modes that are used for IVUS RF signal analysis, including virtual histology-IVUS (VH-IVUS) (Volcano Therapeutics Inc., Rancho Cordova, CA, USA), iMap^TM^ IVUS (Boston Scientific Corp., Marlborough, MA, USA), integrated backscatter IVUS (IB-IVUS) (YD Co., Ltd., Nara, Japan) [35]. A summary of the IVUS and IVUS-based imaging modalities is presented in Table 1.

VH-IVUS is the first commercially available RF signal-based tissue composition analysis tool and is the most widely applied in clinical practice nowadays [35]. VH-IVUS is based on the spectral analysis of the raw backscattered IVUS RF data. A mathematical autoregressive model is adopted to analyze the backscattered RF data; tissue color-coded maps are constructed, which classify plaque into four major categories, including fibrous tissue (green), fibro-fatty (light green), necrotic core (red) and dense calcium (white) [36]. Currently, VH-IVUS can be implemented with either a 20 MHz, 2.9 Fr phased-array transducer catheter or a 45 MHz, 3.2 Fr rotational catheter [22]. iMap^TM^ IVUS utilizes a pattern recognition algorithm to process the spectra of the raw backscattered RF signals that are obtained from a fast Fourier transformation. The tissues are color-coded as four major types, including fibrotic (light green), lipidic (yellow), necrotic (pink) and calcified (blue). IB-IVUS is also based on analyzing the RF signals via a fast Fourier transformation. The color code for tissue types is fibrosis (light green), dense fibrosis (yellow), lipid (blue) and calcified (red). Details about the concept, imaging procedures and applications of different IVUS RF signal-based imaging modalities can be found elsewhere [22,23].

### 2.2. Evaluation Parameters for IVUS Imaging

To evaluate IVUS image quality, three critical factors should be typically taken into consideration, which are spatial resolution, imaging sensitivity and image contrast.

The spatial resolution of an ultrasound image is defined as the minimum distance between two adjacent features that can be differentiated [37]. The higher the spatial resolution, the smaller the distance which can be distinguished. Since the 2D cross-sectional ultrasound image displays both depth into the vessel wall and width across a segment of interest, spatial resolution is further subcategorized into axial resolution and lateral resolution. Axial resolution, also known as depth or longitudinal resolution, is characterized as the capacity to differentiate closely adjacent features along the axis of the ultrasound beam, which can be estimated using [38]

(1) $$R_{axial}=\frac{c}{2f_{c}BW}=\frac{\lambda }{2BW}$$

where *c* is the speed of sound, *f*_c_ is the ultrasound transducer center frequency, *BW* is the −6 dB fractional bandwidth of the ultrasound transducer, *λ* is the wavelength. The typical axial resolution ranges ~70–200 µm for 20–50 MHz ultrasound transducers [39].

Lateral resolution is characterized as the capability to distinguish adjacent features in the direction perpendicular to the propagation direction of the ultrasound beam, which can be estimated as [38]

(2) $$R_{lateral}=\frac{cF_{\#}}{f_{c}}=\lambda F_{\#}$$

where $F_{\#}$ represents the *f*-number, defining as the ratio of focal length to the aperture size of the ultrasound transducer. For an unfocused transducer, the natural focal length $F_{n}$ calculated using Equation (3) can be used to evaluate lateral resolution [40]:

(3) $$F_{n}=\frac{D^{2}}{4\lambda }$$

where *D* is the diameter of the ultrasound transducer. It should be noted that Equation (2) represents the lateral resolution at the focal point and the lateral resolution will downgrade in the off-focus region. For the commonly used IVUS imaging system with transducer frequency ranging 20–50 MHz, the lateral resolution is ~200–250 µm [39].

Another critical image quality index is IVUS imaging sensitivity, which is usually represented by signal-to-noise ratio (*SNR*), illustrating the capability to detect an ultrasound echo above the background electrical and thermal noise [41].

(4) $$SNR=20\log_{10}\frac{V_{tissue}}{V_{noise}}$$

where $V_{tissue}$ is the acoustic signal received from an echogenic region of interest; $V_{noise}$ is the signal received when no ultrasound wave is being transmitted (i.e., an anechoic region). It is noted that *SNR* is a function of imaging penetration depth. With the increase of imaging penetration depth, due to the acoustic attenuation resulting from both scattering and absorption, less acoustic energy will be reflected, causing *SNR* to decrease. In addition, since the acoustic scattering and absorption increase with the ultrasound frequency, lower *SNR* is usually expected at higher frequency. For the IVUS applications, the imaging penetration depth is defined as the depth at which SNR falls below 6 dB. The penetration depth of the existing IVUS system is ~6–12 mm [39].

Image contrast represents the capability to distinguish between a feature of interest and the surrounding tissue signals, which is computed using [42]

(5) $$CR=\frac{\left|\mu_{T}-\mu_{B}\right|}{\sqrt{\sigma_{T}^{2}+\sigma_{B}^{2}}}$$

where $\mu_{T}$ and $\mu_{B}$ are the acoustic signal magnitude in the target and background regions, respectively; $\sigma_{T}$ and $\sigma_{B}$ are the standard deviation of the signal magnitude in each region. Ultrasound image contrast originates from the acoustic impedance differences within the region of interest. It is particularly critical for small features that requires great contrast to be visible against the background speckle.

Other factors that may be used to evaluate an IVUS imaging system include manual versus auto-pullback, IVUS imaging and fluoroscopic angiography co-registration, etc. [43], which are not included in this review since they are not closely related with the IVUS transducer performance.

### 2.3. IVUS Catheter

**Figure 2:**
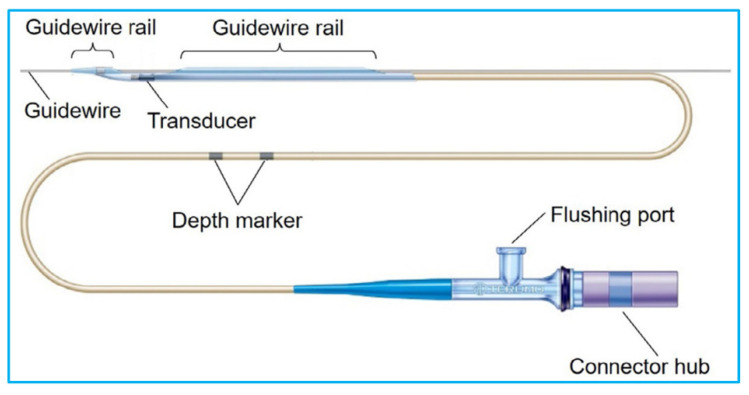
An illustration of an IVUS catheter fed over a guidewire. Reprinted from [45] with permission.

The IVUS catheter is a thin, flexible tube with a miniature transducer mounted on the distal end to image the interior of blood vessels (Figure 2). The proximal end of the catheter connects to a workstation that converts the reflected ultrasound waves from the vessel walls into real-time images to display on a monitor. To conduct IVUS imaging, an IVUS catheter is fed over a guidewire first and angiography is used to guide the catheter to the region of the vessel to be imaged. The IVUS transducer is placed either distal to the region to be imaged and then pulled back through the region of stenosis, or directly placed at the interested area for image acquisition [44].

In the USA, there are two major manufacturers with FDA-cleared IVUS systems, which are Boston Scientific and Philips (after acquisition of Volcano Therapeutics in 2015) [25]. Other top manufacturers include Terumo (Somerset, NJ, USA), Infraredx (Burlington, MA, USA) and ACIST Medical Systems (Eden Prairie, MN, USA). The commercially available IVUS catheters from different manufacturers are listed in Table 2. The catheter sizes range from 2.6–3.5 Fr (0.87–1.17 mm) and can be easily guided through a 5–6 Fr femoral sheath [46]. The typical length of the IVUS catheters is 150 cm and can be used to visualize over 15 cm of a coronary artery. The imaging field of the catheters is about 20 mm, which is sufficient for coronary arteries (average diameter 4–5 mm) imaging [47].

### 2.4. Ultrasound Transducers for IVUS Imaging

**Figure 3:**
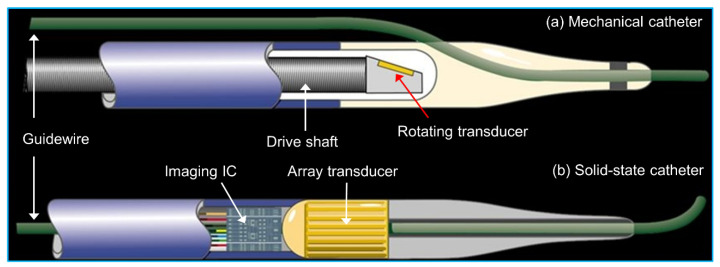
Two different types of IVUS catheters: (**a**) mechanical/rotational catheter with a single-element ultrasound transducer; (**b**) solid-state catheter with a phased-array ultrasound transducer. Reprinted with permission. (Figure courtesy: Boston Scientific, Inc., Marlborough, MA, USA, https://www.bostonscientific.com, accessed on 6 January 2021).

Currently, there are two types of catheters based on the ultrasound transducer structures implemented, which are mechanical/rotational catheter and solid-state catheter (Figure 3) [48]. Both types of catheters can generate a 360° cross-sectional image plane perpendicular to the catheter tip. The major difference between them is the ultrasonic wave transmitting and receiving modes [49]. In the mechanical/rotational catheter, a single-element ultrasound transducer is mounted at the tip of a flexible drive shaft housed in a protective sheath; an external motor drive attached to the proximal end of the catheter rotates the transducer at a speed of 1800 revolutions/min via spinning the drive cable; the transducer transmits and receives the ultrasonic waves at 1° increment to synthesize a cross-sectional image of the vessel [50]. In the solid-state catheter, no rotating components are present. A phased array transducer with 64 transducer elements is mounted circumferentially around the tip of the catheter. The transducer elements are sequentially activated by the integrated circuit in the catheter tip with different time delays to generate an ultrasonic beam that sweeps the circumference of the vessel. The reflected ultrasonic signals from each segment of the vessel wall are collected to reconstruct the cross-sectional image [51]. The comparison of these two types of catheters is shown in Table 3. The most powerful advantage of a solid-state catheter over a mechanical/rotational catheter is that electrical scanning allows for stable imaging, which makes it possible for accurate speckle analysis, and flow analysis and palpography in IVUS imaging.

**Figure 4:**
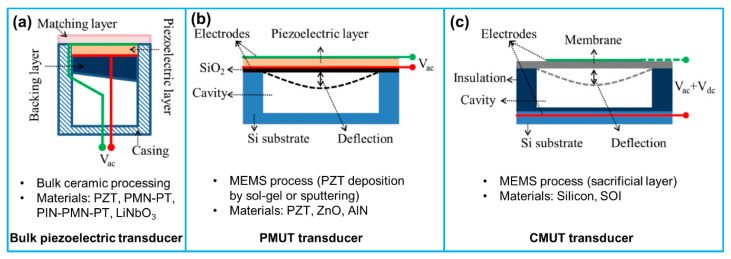
Cross-sectional structures of three different kinds of ultrasound transducers: (**a**) piezoelectric ultrasound transducer; (**b**) piezoelectric micromachined ultrasound transducer (PMUT); (**c**) capacitive micromachined ultrasonic transducer (CMUT). Reprinted from [55] with permission.

Four different categories of ultrasound transducers have been reported for IVUS imaging: conventional piezoelectric ultrasound transducer, piezo-composite micromachined ultrasound transducer (PC-MUT), piezoelectric micromachined ultrasound transducer (PMUT) and capacitive micromachined ultrasound transducer (CMUT) (Figure 4) [52,53,54]. The details about structures and fabrication processes of these four types of ultrasound transducers have been reported by many references. Interested readers are referred to the cited references [52,53,55,56,57].

Until now, due to the mature fabrication techniques, piezoelectric ultrasound transducers dominate the market of IVUS catheters [54]. Based on the different applications (mechanical/rotational catheter and solid-state catheter), they can be categorized into two types: single element and phased array. The most commonly utilized piezoelectric material for the ultrasound transducer is lead zirconate titanate (PZT) ceramics, having a high electromechanical coupling coefficient. Other materials, such as lead magnesium niobate-lead titanate (PMN-PT) single crystals and lead indium niobate-lead magnesium niobate-lead titanate (PIN-PMN-PT) single crystals, that have even higher electromechanical coupling coefficient have also been used for fabricating broadband IVUS transducers. One of the major concerns of these lead-based piezoelectric materials is that they may be harmful to human health for IVUS imaging applications. While some lead-free piezoelectric materials, such as potassium sodium niobate (KNN), have also been adopted by researchers for IVUS transducers, their inferior acoustic and electrical properties make them almost impossible to surpass lead-based materials [58,59]. Since the piezoelectric material-based ultrasound transducer is the mainstream nowadays, the advancement of piezoelectric transducers for IVUS imaging applications will be comprehensively examined in the following section.

Compared with conventional piezoelectric ultrasound transducers, PMUT demonstrates its uniqueness for IVUS imaging. Contrary to conventional piezoelectric ultrasound transducer, PMUT is compatible with complementary metal oxide semiconductor (CMOS), which means that the fabrication process of PMUT can be easily integrated with supporting electronic circuits. Moreover, PMUT demonstrates small form factor, high capacitance and low electrical resistance. Other advantages include its low sensitivity to parasitic capacitance, low loss and high SNR [60,61]. In 2014, Dausch et al. [62] fabricated two rectangular PMUT arrays containing 256 and 512 active elements for an intracardiac ultrasound catheter, which was operated at 5 MHz. The PMUT arrays were fabricated in silicon-on-insulator substrates using PZT. Based on the 3D ultrasound imaging in a swine model using the fabricated 14 Fr catheter, a frame rate of 26 volumes/sec could be obtained in a 60° × 60° volume sector with 10 cm penetration depth; the frame rate would be increased to 31 volumes/sec while the depth reduced to 8 cm. While this was the first publication about intracardiac echocardiography (ICE), seldom studies have reported PMUTs for IVUS imaging applications. Possible reasons are that the existing fabrication techniques cannot achieve high frequency, and the active materials have low electromechanical coupling coefficient, thus making it difficult to satisfy the bandwidth, resolution and sensitivity requirements for IVUS imaging.

Compared to PMUT, CMUT can achieve higher working frequency and broader bandwidth for IVUS imaging [63]. Furthermore, CMUTs are also CMOS-compatible and have small form factor [64]. Currently, three types of CMUTs have been reported for IVUS imaging applications: cylindrical CMUT array [65], phased CMUT array [66,67,68] and dual-ring CMUT array [69,70,71,72]. Zhuang et al. [65] fabricated a 16 × 16-element flexible 2D CMUT array by using a PDMS trench refilling technique. The array with a size of 4 mm × 4 mm demonstrated a resonant frequency of 4.3–5.0 MHz for DC bias voltage of 70–100 V. Due to the flexibility of the CMUT array, it could be wrapped around a 3 Fr catheter tip for side looking IVUS imaging. Xu et al. [66] designed and fabricated four 12-element 1D CMUT phased arrays for cross-sectional imaging of artery. The CMUT array operated with a resonant frequency of around 40 MHz and an aperture of 0.3 mm × 1.0 mm. Four 90° sector images of the artery were obtained based on the four CMUT arrays configuration. In another study, Gurun et al. [72] developed a dual ring CMUT array with 56 transmit elements and 48 receive elements on two concentric annular rings. The dual ring CMUT array had an outer diameter of 1.5 mm and a center hole diameter of 430 µm for guidewire insertion. Based on the image quality testing at 20 MHz, the array showed axial and lateral resolutions of 92 and 251 µm, respectively. A comparison of IVUS imaging qualities using PMUT and CMUT arrays with that of representative piezoelectric transducers is shown in Table 4. Even though CMUTs have demonstrated large bandwidth and electromechanical coupling coefficient for IVUS imaging, a large voltage bias is necessary for achieving high frequency and large sensitivity operations, which increases the risk of dielectric charging and breakdown of the device [73]. Additionally, due to the different acoustic transmitting and receiving requirements, distinct transmission and reception array structures may be needed, thus complicating the design and fabrication [55].

## 3. Piezoelectric Transducers for IVUS Imaging

As summarized in Table 2, the currently available commercial IVUS transducers including both single-element transducer and phased array transducer, have a center frequency ranging from 20–60 MHz. This frequency range typically provides an axial resolution of 20–100 µm, a lateral resolution of 150–250 µm and a penetration depth of 6–15 mm for IVUS imaging [38,75]. Even though the existing IVUS catheters operating at this frequency range are able to assess lumen size, thickness of the vessel wall and lesion location [76], their spatial resolutions are insufficient to evaluate the thickness of fibrous cap (typically <65 µm), a major precursor lesion for vulnerable plaque rupture and acute coronary syndromes [77]. In order to achieve better diagnostic accuracy of coronary artery diseases, tremendous effort has been made over the last two decades to improve IVUS imaging performance by increasing transducer frequency, adopting new transducer designs and piezoelectric materials, as well as combining it with other imaging modalities.

### 3.1. Single Frequency IVUS Transducer

#### 3.1.1. Conventional Piezoelectric IVUS Transducer

Since the IVUS imaging resolution is inversely related to the transducer frequency according to Equations (1) and (2), the most straightforward way to get better imaging resolution is to improve the transducer frequency. Li et al. [78] fabricated an 80 MHz IVUS transducer using a 30 µm-thick PMN-PT free standing film that had high electromechanical coupling coefficient (*k_t_*~0.55) and dielectric constant ($\varepsilon_{r}/\varepsilon_{0}$~4364). The miniature transducer with an aperture of 0.4 mm × 0.4 mm illustrated a −6-dB bandwidth of 65%, and axial and lateral resolutions of 35 µm and 176 µm, respectively. However, the 80 MHz IVUS transducer only had a penetration depth of 2 mm, which could not assess the whole depth of vessel wall (typically >5 mm). To make a tradeoff between the imaging resolution and penetration depth, Sung and Jeong [79] fabricated a 60 MHz transducer with an aperture of 0.49 mm × 0.4 mm using PMN-PT single crystal. The transducer showed a −6 dB bandwidth of 60.2%, which resulted in axial and lateral resolutions of 24.8 µm and 156.1 µm, respectively, and a penetration depth of ~5 mm. More recently, according to the three-matching-layer method that can widen the transducer bandwidth, Ma and Cao [80] reported a 45 MHz IVUS transducer with an aperture of 0.4 mm × 0.5 mm using PMN-PT single crystal. The transducer illustrated a −6 dB bandwidth of 61%, axial and lateral resolutions of 41.6 µm and 214.7 µm, respectively, and a penetration depth of 5 mm. Zhang et al. [81] developed a 40 MHz transducer using high performance PNN-PZT ceramic with an aperture of 0.33 mm × 0.33 mm. The transducer exhibited a −6 dB bandwidth of 79%, and axial and lateral resolutions of 36 µm and 141 µm, respectively.

Another commonly used method to improve transducer resolution is to develop a focal geometry. Fei et al. [82] developed a 35 MHz IVUS transducer with a half-concave structure using PMN-PT single crystal. The 1.2 mm × 1.2 mm transducer showed a −6 dB bandwidth of 54%, and axial and lateral resolutions of 34.5 µm and 392 µm, respectively. Yoon et al. [83] fabricated an angle-focused IVUS transducer using PMN-PT single crystal with a viewing angle of 60° and center frequency of 45 MHz. The focused transducer illustrated a −6 dB bandwidth of 72%; the axial and lateral resolutions were calculated as 25 µm and 120 µm, respectively. In another study, Lee et al. [84] developed an oblong shaped focused IVUS transducer using PZT ceramic, having a center frequency of 50 MHz and a focal distance of 3 mm. The 0.5 mm × 1.0 mm oblong shaped transducer showed a −6 dB bandwidth of 57% and a lateral resolution of 150 µm.

While lead-based piezoelectric materials including PZT ceramic, PMN-PT and PIN-PMN-PT single crystal are the most popularly adopted materials for IVUS transducers due to their excellent piezoelectric performance and mature fabrication techniques, the raised health and environment concerns about the toxic lead-based materials have inspired researchers to develop lead-free piezoelectric materials for IVUS transducers [61,85,86]. Yan et al. [87] fabricated a 30 MHz IVUS transducer with an aperture of 0.8 mm × 0.8 mm using lead-free BZT-50BCT ceramic that showed a high $\varepsilon_{r}/\varepsilon_{0}$ of ~2800 and piezoelectric coefficient *d*_33_ of ~600 pC/N. The fabricated transducer demonstrated a −6 dB bandwidth of 53%. Zhu et al. [88] utilized 35 µm Li doped KNN (KNLN) thick film for developing a 50 MHz side-looking IVUS transducer, having an aperture of 0.4 mm × 0.4 mm. Similar to lead-based materials, *k_t_* of the thick film was 0.44; the fabricated transducer illustrated a −6 dB bandwidth of 61.5%. Fei et al. [89] adopted lithium niobate (LiNbO_3_) to develop single element transducers with ultrahigh frequencies (100–300 MHz) for super-resolution ultrasound imaging applications. While the axial and lateral resolutions of 15.4 µm and 16.4 µm, respectively could be obtained for the 100 MHz focused transducer, the high attenuation in blood and poor penetration depth have limited its applications in IVUS imaging.

#### 3.1.2. Piezo-Composite Micromachined Ultrasound Transducer (PC-MUT)

**Figure 5:**
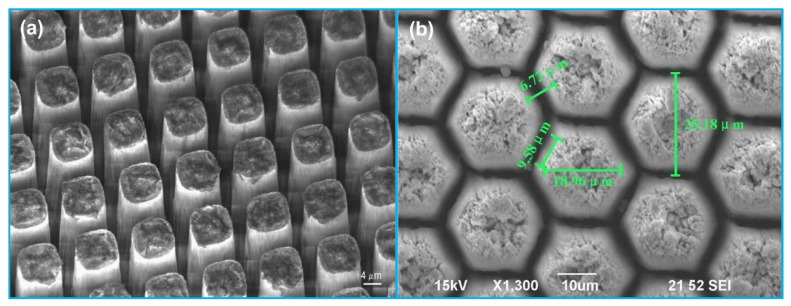
SEM images of pillar arrays for a piezo-composite micromachined ultrasound transducer (PC-MUT). (**a**) PMN-PT single crystal pillars for a 60 MHz PC-MUT. Reprinted from [94] with permission. (**b**) PZT-5H ceramic hexagonal pillars for a 50 MHz PC-MUT. Reprinted from [100] with permission.

An alternative method to improve imaging resolution without sacrificing penetration depth is to develop a transducer with broader bandwidth at relatively lower frequency. Compared with monolithic piezoelectric materials, 1-3 piezo-composite structure can provide broader bandwidth due to its higher *k_t_*. Moreover, the acoustic impedance of a piezo-composite is much lower than that of a bulk piezoelectric material, which can significantly reduce the acoustic mismatch between transducer and tissue [52]. However, developing a high frequency 1-3 piezo-composite using the conventional dice-and-fill technique usually provides a low volume fraction, and a large pillar and kerf width to thickness aspect ratio due to the limitation of dicing blade width [90]. The minimum existing blade width is typically 10–15 µm, thus limiting the transducer frequency <20 MHz [52]. In addition, due to the limitation of current dicing capability, the shape of pillars in a 1-3 piezo-composite is squarely distributed. Nevertheless, studies have reported that other shape of pillars such as hexagonal can achieve better electromechanical and acoustic performance [91,92]. In order to overcome the limitations of conventional dice-and-fill method, Jiang et al. [52,93] developed photolithography-based deep reactive ion etching (DRIE) micromachining process for fabricating high frequency 1-3 piezo-composite, namely PC-MUT (Figure 5a). The PC-MUT technology takes advantages of high *k_t_* of PMN-PT single crystals, fine patterning features of photolithography and DRIE for fabrication of composite microstructures. Based on the PC-MUT technology, high frequency (20–100 MHz) 1-3 PMN-PT composite transducers with a post width of 12–14 µm, a kerf width of 3–4 µm and a *k_t_* of 0.72 were fabricated for IVUS imaging applications [94,95]. Following these works, Li et al. [96] reported a 40 MHz miniature transducer with an aperture of 0.5 mm × 0.4 mm fabricated by PIN-PMN-PT single crystal 1-3 composite. Compared with commonly used piezoelectric materials for an IVUS transducer, such as PZT ceramic and PMN-PT single crystal, PIN-PMN-PT single crystal does not only have high *k_t_* and $\varepsilon_{r}/\varepsilon_{0}$, but also illustrate improved electrical and thermal stability [97,98,99]. Based on the authors’ results, an effective electromechanical coupling coefficient of 0.75–0.78 was obtained; the fabricated 40 MHz transducer demonstrated a −6-dB bandwidth of 86%, resulting in axial and lateral resolutions of 43 µm and 226 µm, respectively.

More recently, a cold ablation process that is based on focused picosecond UV laser is also reported for high frequency 1-3 piezo-composite fabrication (Figure 5b). Since the pulse width of picosecond laser is shorter than the thermal relaxation time, the cold ablation process can be used to remove thin material layers without significant thermal side-effects [100]. Xu et al. [101] fabricated a 45 MHz 1-3 PZT-5H composite using a cold ablation process with a picosecond UV laser. The width of the pillar was 20.5 µm; the kerf of the pillar was 4.5 µm; the fabricated piezo-composite demonstrated a *k_t_* of 0.73. In another work, Li et al. [100] fabricated a 50 MHz 1-3 PZT-5H composite with a hexagonal pillar geometry using the cold ablation technique. The kerf width of the 1-3 piezo-composite was 5 µm; the pillar width was 10 µm, showing a PZT-5H volume fraction of 64%. The 1-3 piezo-composite demonstrated a *k_t_* of 0.7 and a −6 dB bandwidth of 68.8%.

#### 3.1.3. Micromotor Driven IVUS Imaging

Currently, all the mechanical/rotational IVUS catheters obtain the vessel cross-sectional images via a rotating reflector driven by a proximal motor and a flexible driving shaft [102]. The major limitation of this type of configuration is the image distortion, known as non-uniform rotation distortion (NURD), that occurs when the catheter passes through a bending vessel due to the friction force between the flexible drive shaft and catheter wall [103]. To avoid NURD, a different catheter structure with the driven motor placed on the distal end of catheter has been proposed and developed for IVUS imaging, thus directly rotating an ultrasonic transducer or a miniature reflecting mirror instead of transmitting the rotational motion by a flexible shaft. Peng et al. [103] developed a three-phased synchronous electromagnetic micromotor, which had a dimension of 1.2 mm × 3.7 mm and a maximum rotating speed of 16,500 rpm. At the driving frequency of 10 Hz, the maximum angular error was 4°. In addition to electromagnetic micromotor, piezoelectric micromotor was also reported [104,105,106,107,108]. For example, Zhang et al. [109] designed and fabricated a piezoelectric micromotor with a size of 1 mm × 10 mm and a maximum rotating speed of 6450 rpm. The speed of the micromotor was controllable and the maximum angular error was 8°, which would not cause obvious image distortion.

The imaging performance comparison of the reported single frequency IVUS transducers is summarized in Table 5.

### 3.2. Dual Frequency IVUS Transducer

**Figure 6:**
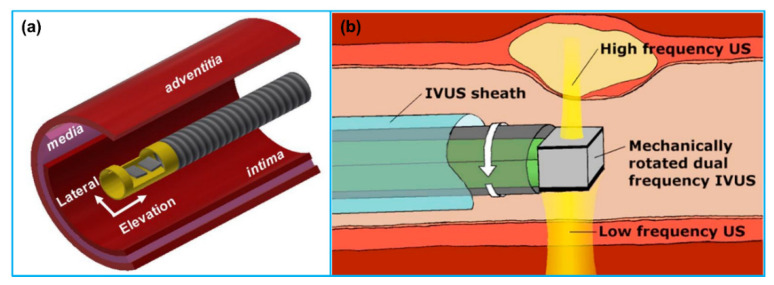
(**a**) An illustration of side-by-side transducer configuration for a dual frequency IVUS transducer. Reprinted from [114] with permission. (**b**) An illustration of back-to-back configuration for a dual frequency IVUS transducer. Reprinted from [111] with permission.

Even though various high frequency IVUS transducers have been reported with an enhanced axial resolution ranging 25–40 µm, their penetration depth is limited by the greater acoustic attenuation at higher frequencies. For the clinical applications, the desired IVUS imaging performance is a spatial resolution high enough to evaluate the thickness of fibrous cap (typically <65 µm) and a penetration depth >5 mm [110]. However, these two requirements cannot be met at the same time since the penetration depth is reduced due to the frequency-dependent acoustic attenuation while the transducer frequency is increased to achieve the desired spatial resolution. To overcome the inherent tradeoff between the acoustic attenuation and spatial resolution, a dual frequency transducer structure has been reported [74,111]. Two IVUS transducers with different frequencies are placed in catheter; the transducer with lower frequency (20–40 MHz) ensures large penetration depth while the other transducer with higher frequency (80–150 MHz) provides high resolution for superficial microstructure imaging. The configuration of these two transducers can be categorized into two types: side-by-side [112,113] and back-to-back [74,111]. In side-by-side configuration (Figure 6a), two IVUS transducers are arranged axially along the catheter with a center-to-center spacing of ~1–3 mm; the IVUS image co-registration is performed via pull-back scanning of the catheter. In back-to-back configuration (Figure 6b), two transducers are aligned bidirectionally in the thickness direction; the image co-registration is accomplished by rotating the catheter by 180°. A comparison of the reported dual frequency IVUS transducers for imaging is illustrated in Table 6.

### 3.3. Multifrequency IVUS Imaging

Tissue harmonic imaging, a widely used technique in commercial ultrasound systems to simultaneously improve the spatial and contrast resolutions of ultrasound images, has also been reported to enhance the spatial resolution of IVUS images while maintaining penetration depth. While ultrasound waves propagate into the human tissue, the harmonic components of the transmitted ultrasound waves are generated caused by the nonlinear nature of biological media [117]. It has been shown that the energy of harmonics is nonlinearly dependent on the transmitted acoustic pressure; in particular, the energy of second harmonic is proportional to the square of transmitted acoustic pressure [118]. It means that harmonics are mainly generated from the energy of the main lobe in the transmitted beam profile. Due to the low energy levels of the side and grating lobes in the harmonic beam profile, enhanced contrast resolution can be achieved [119,120]. Moreover, compared to the transmitted beam profile, the main lobe width of a harmonic beam profile is narrower; it reduces as the harmonic order increases [121]. Thus, compared with fundamental ultrasound imaging, tissue harmonic imaging can provide a higher spatial resolution; the resolution will also increase with the rise of harmonic order. Currently, the second harmonic imaging is adopted in most of ultrasound imaging systems [120], in which an ultrasound transducer is employed for both transmitting fundamental ultrasound waves and receiving second harmonic waves. For this end, an ultrasound transducer with a −6 dB bandwidth > 70% should be developed [115]. Even though using piezoelectric 1-3 composite for transducer fabrication can achieve a bandwidth > 70% at the expense of transmitting ultrasound pressure as a result of lowering mechanical quality factor [95,122], it is challenging to fabricate a miniature IVUS transducer with high frequency and desired structure [123,124]. Considering this perspective, dual frequency transducers with two different center frequencies will be the best choice for high frequency tissue harmonic imaging.

**Figure 7:**
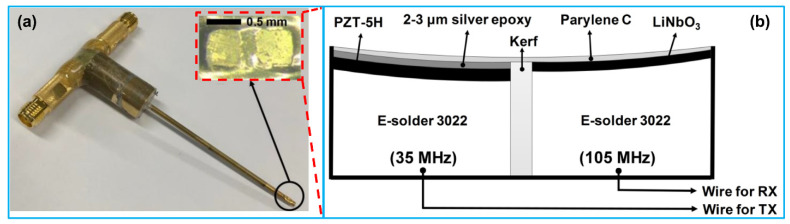
Dual frequency focused transducer for IVUS imaging using harmonics: (**a**) a photograph image of the fabricated transducer; (**b**) structure of the dual frequency focused transducer. Reprinted from [114] with permission.

While the side-by-side configuration of dual frequency transducers is applicable for tissue harmonic imaging, the simple arrangement of two transducer elements cannot work well since the harmonic signals cannot receive effectively resulting from the different focal depths of the two elements. Recently, Lee et al. [110] developed a three-element, dual frequency IVUS transducer for tissue harmonic imaging. The three elements were arranged side by side in the horizontal direction and formed a spherical shape. Based on their phantom imaging results, the tissue harmonic imaging demonstrated higher spatial resolution and imaging contrast as well as lager imaging depth than that of the 70 MHz fundamental imaging. Following this work, Lee et al. [115] fabricated a dual-element, dual frequency IVUS transducer for harmonic imaging; one element with a center frequency of 35 MHz was used as transmitter, and the other element with a center frequency of 70 MHz was used for receiving the second harmonic signals. In addition, Lee et al. [114] developed a dual-element, dual frequency IVUS transducer for the third harmonic imaging as well (Figure 7). The two elements were also arranged side-by-side and spherically shaped with a radius of 2.5 mm. One element having a center frequency of 35 MHz was used for ultrasound transmission and the other element having a center frequency of 105 MHz was used for receiving the third harmonic signals. The phantom imaging results showed that the produced third harmonic images had higher spatial resolution and larger penetration depth than the fundamental images. The comparison of the spatial resolution of harmonic image with that of fundamental image are showed in Table 7.

**Figure 8:**
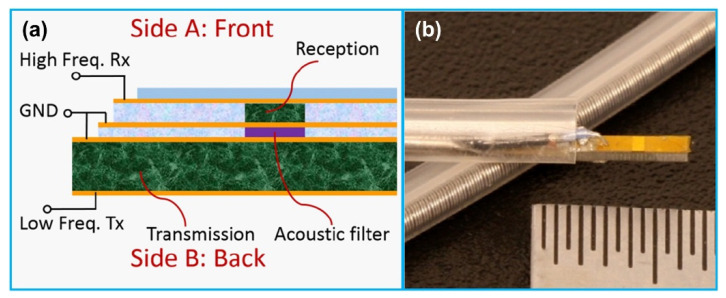
Stack dual frequency transducer for superharmonic IVUS imaging. (**a**) Schematic illustration of the dual frequency transducer structure; (**b**) A photograph image to show the fabricated transducer mounted inside a commercial catheter sheath. Reprinted from [136] with permission.

Dual frequency IVUS transducers for superharmonic imaging with ultrasound contrast agents were also developed [125,126,127]. The two elements of the transducer were stacked vertically with co-aligned transmit and receive beams and electrically separated by a frequency selective isolation layer between them (Figure 8). The lower layer element having a low center frequency of 2–10 MHz transmitted acoustic waves to oscillate contrast agents; the upper layer element having a high center frequency of 10–30 MHz received superharmonic signals generated by the contrast agents. This kind of contrast enhanced IVUS imaging technique has been successfully applied for imaging vas vasorum, a microvasculature with a diameter < 200 µm that is closely related to early atherosclerotic plaque [128,129]. For example, Ma et al. [125,130] developed a dual frequency (6.5/30 MHz) IVUS transducer to excite microbubbles near their resonance and detect their superharmonic vibrations. The lower layer 6.5 MHz element had an aperture of 0.6 mm × 3 mm; the upper layer 30 MHz element had an aperture of 0.6 mm × 0.5 mm. The receiving element demonstrated a −6 dB bandwidth of ~60%, illustrating a broadband microbubble response. Based on the phantom imaging results, a high contrast-to-tissue ratio of 12 dB and axial resolution of 200 µm were achieved. Following that work, several other dual frequency IVUS transducers for microbubble contrast agent imaging have also been reported by the group [131,132,133]. Martin et al. [134] developed a dual frequency (5.5/37 MHz) IVUS transducer for visualizing contrast flow in micro-vessels. While the B-mode imaging showed a slightly higher contrast enhancement compared with the dual frequency mode, the dual frequency mode illustrated the capability of suppressing the tissue harmonics effectively with a lower tissue-to-noise ratio. The isolation layer or the acoustic filter between the transmitting and receiving element is a critical component to control wave propagation in multi-frequency system. An acoustic filter is mainly utilized to prevent the high-frequency wave from backward propagation while passing the forward low-frequency wave efficiently. Ma et al. [135] explored the acoustic filter design criteria according to the microwave transmission line theory. Based on their design, the acoustic filter layer was proved to suppress the high frequency aliasing echo by 14.5 dB and amplify the low frequency transmission by 8.0 dB, increasing an axial resolution from 416 µm to 86 µm in imaging. In addition, in order to optimize the dual frequency transducer design for the contrast enhanced IVUS imaging, Ma et al. [136] studied the impact of IVUS transducer layouts, transmitting frequencies and active materials on the imaging performance. They found that the stacked configuration showed the advantage over the other transducer configurations in the uniformity of the transmitting beam profile without a drop of pressure near the center of the transducer, where the receiving element had the highest sensitivity. In addition to the dual frequency IVUS transducer development, in order to obtain high quality IVUS contrast images, new signal processing method was proposed [137] and an integrated IVUS contrast imaging system was developed for in-vitro phantom imaging tests [138].

### 3.4. Array for IVUS Imaging

More recently, piezoelectric array transducer has also emerged for IVUS imaging applications. Compared with single element IVUS transducers, IVUS array transducers demonstrate unique features. Unlike the single element transducers for mechanical/rotational catheters, the solid-state catheter integrated with an IVUS array transducer can get rid of the non-uniform rotational displacements and the off-axis errors due to its stationary design. Furthermore, an IVUS array transducer can be adopted with beamforming during transmitting and receiving, which can increase the frame rates and decrease the point spread functions, thus optimizing the image quality.

#### 3.4.1. Single Frequency Array

**Figure 9:**
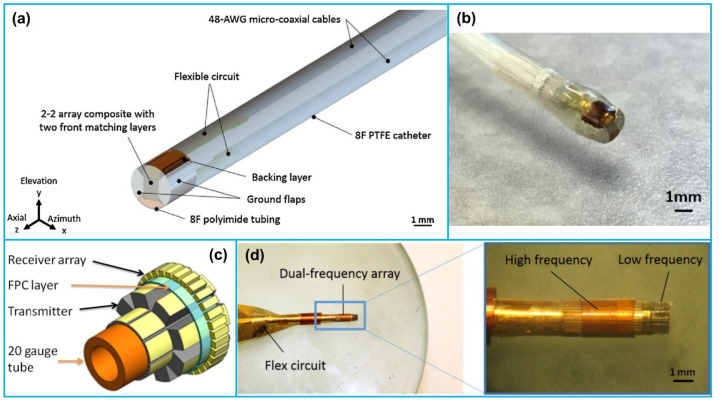
A 30 MHz forward-looking phased array transducer for IVUS imaging: (**a**) schematic of the array structure enclosed in an 8 Fr catheter; (**b**) photograph image of the packaged phased array transducer. Reprinted from [140] with permission. A dual frequency cylindrical array for contrast enhanced IVUS imaging: (**c**) illustration of the dual frequency cylindrical array structure; (**d**) photograph images of the fabricated dual frequency cylindrical array. Reprinted from [143] with permission.

Cabrera-Munoz et al. [140] reported a 30 MHz forward-looking phased array transducer which was composed of 32-element of 2-2 PMN-PT composite for IVUS imaging. The array transducer had a dimension of 0.8 mm × 1 mm, and a natural focal depth of 5 mm. The phantom imaging results demonstrated that the array showed a −6 dB bandwidth of 36.4%, and axial and lateral resolutions of 65 µm and 215 µm, respectively. Based on the porcine carotid artery phantom imaging evaluation results, the array provided a penetration depth > 5 mm for IVUS imaging. Following this work, Cabrera-Munoz et al. [141] fabricated a 15 MHz side-looking phased array transducer as well, which was composed of 64-element of 2-2 PMN-PT composite. The fabricated array showed an aperture of 3.2 mm × 1.8 mm, which could be integrated into a 10 Fr catheter. The phantom imaging results demonstrated that the array illustrated axial and lateral resolutions of 90 µm and 420 µm, respectively, and an imaging penetration depth > 8 mm. In another work, Li et al. [142] developed a 40 MHz circular array with micromachined PMN-PT 1-3 composite elements for IVUS imaging (Figure 9a,b). The circular array consisted of 50 elements with a pitch of 100 µm around a needle, having an outer diameter of 1.7 mm. Using the deep reactive ion etching method, the PMN-PT 1-3 composite was fabricated with a pillar diameter of 18 µm and a kerf of 3 µm. The developed array demonstrated a center frequency of ~39 MHz and a −6 dB bandwidth of ~82%. In vitro phantom imaging results demonstrated that the circular array had an axial resolution of 60 µm with a penetration depth of 3 mm, and a dynamic range of 30 dB.

#### 3.4.2. Dual Frequency Array

More recently, Wang et al. [143,144] reported a dual frequency IVUS cylindrical array with a reduced form-factor lateral mode transmitter (2.25 MHz) and a high frequency receiver (30 MHz) for contrast enhanced IVUS imaging (Figure 9c,d). The low-frequency transmitter contained 8 PMN-PT elements with a pitch size of 650 µm; the high-frequency receiver contained 32 PMN-PT elements with a pitch size of 160 µm. In vitro superharmonic imaging of a 200 µm tube showed that the axial resolution of the dual frequency array was 162 µm with a contrast-to-noise ratio of 16.6 dB. Wu et al. [145] fabricated a dual frequency (7/35 MHz) circular array for contrast enhanced IVUS imaging as well. The 7 MHz transmit array was composed of 8 PZT-5H 1-3 composite elements with a pitch of 200 µm and pillar dimension of 160 µm; the 35 MHz receive array consisted of 32 PMN-PT 1-3 composite elements with a kerf of 3 µm and pillar diameter of 18 µm. The dual frequency circular array was wrapped around a 1.2 mm-diameter needle for IVUS imaging. Their characterization results showed that the average −6 dB bandwidth of the receiving elements was ~68%, demonstrating a broad bandwidth for detecting microbubble response.

## 4. IVUS-Based Multimodality Intravascular Imaging

Even though IVUS imaging can provide the cross-sectional visualization of the coronary artery wall and the quantitative evaluation of the lumen size and plaque characteristics [146,147], its intrinsic limitations including the low spatial resolution and considerable noise hinder the detailed assessment of plaque composition and visualization of microfeatures of plaque that are associated with increased vulnerability [148,149,150]. In order to address these limitations and provide a complete assessment of coronary artery, over the past two decades, alternative intravascular imaging techniques, including optical coherence tomography (OCT) [151,152], near-infrared spectroscopic (NIRS) imaging [153,154], intravascular photoacoustic (IVPA) imaging [155,156], near infrared fluorescence (NIRF) imaging [157,158], time resolved fluorescence spectroscopic (TRFS) imaging [159,160] and fluorescence lifetime imaging (FLIM) [161,162], have been emerged as a result of the miniaturization of medical devices and advances in image processing. Since the working principles of these intravascular imaging modalities have been thoroughly reviewed elsewhere, this review will concentrate on their intravascular coronary imaging applications.

As the optical analogue of IVUS, OCT is an emerging imaging modality that uses low coherence light to perform cross-sectional imaging of the arterial wall with a high resolution of 1–15 µm [163]. Similar to IVUS, during intravascular imaging process, an OCT catheter with an imaging core at the distal end is firstly advanced into the region of interest. Blood needs to be removed from the region of interest by flushing with contrast or saline or using an occlusive method [164]. The catheter is then automatically pulled back with a speed of 1–25 mm/s as it images the vessel. Due to its superior resolution, the presence of neo-vessels and micro-calcifications, and the thickness of fibrous cap can be visualized [165]. However, the key limitation of OCT is its low penetration depth of 0.5–2 mm that is not sufficient to visualize the entire vessel wall and discriminate lipid from calcific tissue. Other limitation is that it requires temporal clearance of blood by using saline flushes or an occlusive balloon to overcome the blood interference problem [164].

NIRS, a spectroscopic technique that has been commonly used in industries for both qualitative and quantitative measurements of chemicals, has been commercialized for the study of coronary atherosclerosis and plaque composition [166,167]. Since different organic molecules absorb and scatter the near-infrared light at different degrees and wavelengths [168], the chemical composition of plaque can be obtained by analyzing the spectrum of the scattered light. Similar to IVUS and OCT imaging, a catheter is advanced into the artery of interest and cross-sectional images of the arterial segment are obtained via withdrawing the catheter. A color map is assigned to the image, known as chemogram, which indicates the probability of the presence of lipid-rich plaques in each region [169]. Even though NIRS imaging provides a reliable and quantitative assessment of lipid-core plaques, it cannot assess lumen size, vessel wall dimension and plaque burden. In addition, it cannot provide information about the depth of the lipid component into the vessel wall [21].

IVPA imaging that is based on localized thermal expansion, uses a nanosecond laser to excite biological tissues to generate acoustic waves [155,156]. The generated acoustic waves propagate through tissues and are received by an ultrasound transducer. The acoustic signal strength is proportional to the absorption coefficient of the tissue as well as the light intensity. Based on the tissue absorption coefficient which is related to the chemical composition of the tissue, IVPA image permits differentiation of the tissue types. The photoacoustic excitation wavelength can be selected to generate absorption contrast between the relevant components in the vessel wall and plaque, such as collagen, calcified tissue and lipids, allowing characterization of plaque composition [170,171,172]. While IVPA imaging provides high-detailed information about plaque chemical composition, it cannot provide any information about lumen size, vessel wall and plaque dimensions, as well as plaque distribution. Moreover, similar to optical intravascular imaging techniques, it requires blood clearance to get high quality image [173].

NIRF imaging is an emerging cellular and molecular imaging technique that uses laser induced NIR signal to stimulate NIRF emission of vessel wall and plaques that have injected with fluorescence-imaging agents [174]. It has been demonstrated that the activity of plaque inflammation can be detected by injecting an imaging contrast agent to the sites of the inflamed tissues [175]. The limitations of NIRF imaging are its inability to assess lumen size, vessel wall and plaque dimensions and compositions, as well as provide depth information. In addition, it requires injection of an activatable agent [176].

TRFS and FLIM are the extensions of fluorescence spectroscopy [177]. They are used to monitor the change in fluorescence over time (picoseconds to milliseconds) of a sample when irradiated with UV, visible or NIR light [178]. Since structural proteins and lipid constituents in atherosclerotic plaques have distinct fluorescence properties, changes in plaque composition can be evaluated through analysis of various fluorescence-derived parameters such as intensity, spectra or lifetime values [179]. Studies have reported that the application of these fluorescence techniques can assess plaques with TCFA and discriminate of lipid-rich and inflamed tissues [180]. The major limitations of TRFS and FLIM include their inability to assess lumen size, vessel wall morphology and dimension, as well as plaque burden. The comparison of different intravascular imaging techniques is summarized in Table 8.

**Figure 10:**
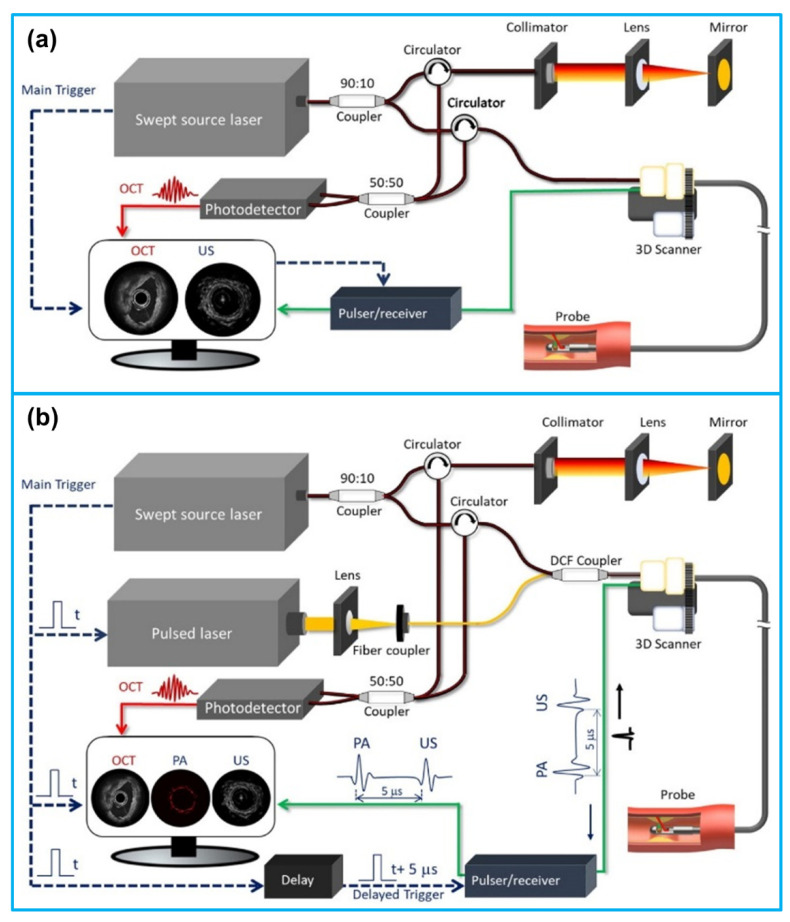
Schematic illustration of multi-modality intravascular imaging systems. (**a**) Dual-modality IVUS-OCT imaging system; (**b**) tri-modality IVUS-OCT-IVPA imaging system. Reprinted from [184] with permission.

While each intravascular imaging modality has unique features that supply critical information about the extent and severity of atherosclerosis, they also possess inherent limitations that prevent complete evaluation of the coronary arteries. To address this challenge, multi-modality intravascular imaging that combines different imaging techniques with complementary strengths into advanced images has been proposed and developed. Since the multimodal intravascular imaging technology has been reviewed extensively by many researchers [21,181,182,183,184], in this section, we concentrate on the reported applications of IVUS-based dual-modality intravascular imaging including IVUS-OCT, IVUS-NIRS, IVUS-IVPA, IVUS-NIRF, IVUS-TRFS (FLIM) and tri-modality intravascular imaging including IVUS-OCT-NIRF and IVUS-OCT-IVPA. Representative dual-modality and tri-modality intravascular imaging systems are demonstrated in Figure 10. The comparison of these multi-modality intravascular imaging techniques for evaluation of vulnerable plaques is illustrated in Table 9. Currently, both IVUS-OCT and IVUS-NIRS imaging catheter systems are commercially available. Other multi-modality intravascular imaging systems have been developed and tested ex vivo and pre-clinical in vivo settings. A summary of the reported multi-modality intravascular imaging catheters is shown in Table 10.

## 5. Conclusions and Perspectives

In this paper, the recent advances in ultrasound transducers for IVUS imaging were comprehensively reviewed. The basic IVUS imaging principle and the recently emerged IVUS image processing techniques were firstly presented. Three major evaluation parameters including spatial resolution, imaging sensitivity and image contrast for IVUS imaging were then summarized. Afterwards, two types of IVUS catheters including mechanical/rotational catheter and solid-state catheter were reviewed. Following that, three types of ultrasound transducers (piezoelectric ultrasound transducer, PMUT, CMUT) for IVUS imaging were examined in detail. In particular, the recent development of piezoelectric ultrasound transducers for IVUS imaging was extensively reviewed based on their working mechanisms, configurations and active materials adopted. Finally, the IVUS-based multimodality intravascular imaging was summarized and compared.

Even though IVUS imaging has become an established technique in clinical settings, its relatively low resolution made it difficult to accurately measure the fibrous cap thickness. In order to achieve both high resolution for detecting superficial plaque features and large penetration depth for visualizing overall morphology of vessel wall, high frequency ultrasound transducer based on PC-MUT technology has been developed, which illustrates the promise for clinical applications. Another area of active exploration is to develop dual frequency IVUS transducers. While it has been shown that the low frequency element could achieve an imaging capability similar to that of existing commercial catheters, and the high frequency element improved the axial resolution at the cost of reduced penetration depth, the feasibility of the dual frequency IVUS transducer needs to be further validated in a more clinically relevant catheter design. Moreover, while the in vitro phantom imaging results of both multifrequency IVUS imaging and array imaging were promising, in vivo imaging of atherosclerotic plaques has not been conducted with the developed transducers. Considering that each intravascular imaging modality has its own unique strengths and limitations, further integration of two or more imaging modalities to supplement each other’s weaknesses will advance the characterization of vulnerable plaques and aid in atherosclerosis diagnosis. In addition, further miniaturizing of the multimodality intravascular catheter will be favorable while delivering such catheter into the complex and confined coronary circulation system.

## Figures and Tables

**Table 1:** A summary of IVUS and IVUS-based imaging modalities.

	IVUS	VH-IVUS	iMap^TM^	IB-IVUS
Type of device	Mechanical and electrical	Mechanical and electrical	Mechanical	Mechanical
Transducer frequency	20–60 MHz	20–45 MHz	40 MHz	40 MHz
Color code	Grayscale	Fibrous: green Fibro-fatty: light green Necrotic core: red Dense calcium: white	Fibrotic: light green Lipidic: yellow Necrotic: pink Calcified: blue	Fibrosis: light green Dense fibrosis: yellow Lipid: blue Calcified: red
Backscatter radiofrequency signal analysis	Amplitude (dB)	Autoregressive model	Fast Fourier Transformation	Fast Fourier Transformation

**Table 2:** The existing available commercial IVUS catheters.

Manufacturer	Product Name	Transducer Frequency	Distal Shaft Profile	Proximal Shaft Profile	Transducer to Tip Length	Axial Resolution	Transducer Type
Boston scientific	OptiCross^TM^	40 MHz	2.6 Fr	3.1 Fr	20 mm	38 µm	Rotational
	OptiCross^TM^ 6	40 MHz	2.9 Fr	3.1 Fr	20 mm	38 µm	Rotational
	OptiCross^TM^ 18	30 MHz	2.9 Fr	3.5 Fr	20 mm	N/A	Rotational
	OptiCross^TM^ 35	15 MHz	6 Fr	8 Fr	10 mm	N/A	Rotational
	OptiCross^TM^ HD	60 MHz	2.6 Fr	3.1 Fr	20 mm	22 µm	Rotational
Philip (Volcano)	Eagle Eye^®^	20 MHz	3.3 Fr	2.9 Fr	10 mm	<170 µm	Phased array
	Revolution^®^	45 MHz	3.2 Fr	3.5 Fr	30 mm	50 µm	Rotational
	Refinity^®^	45 MHz	3.0 Fr	3.0 Fr	20.5 mm	50 µm	Rotational
Terumo	View IT^®^	40 MHz	2.6 Fr	3.2 Fr	29 mm	69 µm	Rotational
	AltaView^®^	60 MHz	2.6 Fr	3.2 Fr	24 mm	<30 µm	Rotational
	AnteOwl WR^®^	40 MHz	2.6 Fr	3.1 Fr	8 mm	N/A	Rotational
	Navifocus WR^®^	40 MHz	2.5 Fr	3.1 Fr	9 mm	N/A	Rotational
	Intrafocus WR^®^	40 MHz	2.8 Fr	3.2 Fr	30 mm	N/A	Rotational
Infraredx	Dualpro^TM^	50 MHz	3.2 Fr	3.6 Fr	20 mm	40 µm	Rotational
ACIST	Kodama^®^	40/60 MHz	3.2 Fr	3.6 Fr	20 mm	40 µm	Rotational

**Table 3:** A comparison of mechanical/rotational catheter and solid-state catheter.

Type	Principle	Feature	IVUS Image Quality	Advantage	Disadvantage
Mechanical/rotational catheter	A single element ultrasound transducer rotates mechanically inside an echolucent distal sheath.	Single element transducer (40–60 MHz) Stabilized pullback trajectory due to the outer sheath	Higher image resolution due to the higher frequencies and larger effective aperture size.	Excellent near-field resolution and no need for digital subtraction	Guidewire artifact due to the guidewire running outside the catheter Air bubble artifact due to the insufficient saline flush Nonuniform rotational distortion
Solid-state catheter	A phased-array ultrasound transducer is activated sequentially in a circular way.	64 elements phased array (20 MHz) Short transducer to tip distance	Larger scanning depth due to the lower ultrasound frequency.	No guidewire artifact due to the guidewire lumen located inside the catheter No nonuniform rotational distortion Stable imaging, allowing for accurate speckle analysis, flow analysis and palpography	Ring down artifact (bright halos surrounding the catheter) due to the poor near-field resolution

**Table 4:** A summary of IVUS imaging qualities using PMUT and CMUT compared with piezoelectric ultrasound transducers.

Ultrasound Transducer	Transducer Type	Frequency	Aperture Size	Penetration Depth	Axial Resolution	Lateral Resolution
PMUT	2D array [62]	5 MHz	1.1 mm × 6.3 mm	30 mm	500 µm	1 mm
CMUT	1D array [66]	35.6 MHz	0.3 mm × 1.0 mm	2.4 mm	N/A	277 µm
	1D array [68]	20.8 MHz	Diameter 2.97 mm	16 mm	55 µm	0.035 rad
	1D array [68]	5 MHz	Diameter 2.97 mm	71 mm	440 µm	0.12 rad
	2D dual-ring array [72]	20.1 MHz	Outer diameter 1.4 mm	4–8.2 mm	92 µm	251 µm
Piezoelectric transducer	Single element [74]	30 MHz	0.5 mm × 0.5 mm	5 mm	46.0 µm	231.5 µm
		90 MHz		2 mm	21.5 µm	123.5 µm
		120 MHz		1 mm	25.7 µm	105.3 µm
		150 MHz		0.5 mm	17.2 µm	87.3 µm

**Table 5:** IVUS imaging performance comparison of the reported single frequency transducers.

Piezoelectric Material	Aperture Size	Frequency	−6 dB Bandwidth	Penetration Depth	Axial Resolution	Lateral Resolution
PMN-PT [78]	0.4 mm × 0.4 mm	80 MHz	65%	2 mm	35 µm	176 µm
PMN-PT [79]	0.49 mm × 0.4 mm	60 MHz	60.2%	~5 mm	24.8 µm	156.1 µm
PMN-PT [80]	0.4 mm × 0.5 mm	45 MHz	61%	5 mm	41.6 µm	214.7 µm
PNN-PZT [81]	0.33 mm × 0.33 mm	40 MHz	79%	N/A	36 µm	141 µm
PMN-PT [82]	1.2 mm × 1.2 mm Focused	35 MHz	54%	N/A	34.5 µm	392 µm
PMN-PT [83]	0.57 mm × 0.57 mm 60° Focused	45 MHz	72%	N/A	25 µm	120 µm
PZT [84]	0.5 mm × 1.0 mm Focused	50 MHz	57%	N/A	N/A	150 µm
BZT-50BCT [87]	0.8 mm × 0.8 mm	30 MHz	53%	N/A	N/A	N/A
Li doped KNN [88]	0.4 mm × 0.4 mm	50 MHz	61.5%	N/A	N/A	N/A
PIN-PMN-PT 1-3 composite [96]	0.5 mm × 0.4 mm	40 MHz	86%	N/A	43 µm	226 µm
PZT-5H 1-3 composite [100]	0.5 mm × 0.6 mm	50 MHz	68.8%	N/A	22 µm	N/A
PZT-5H 1-3 composite [101]	0.5 mm × 0.6 mm	50 MHz	56.9%	N/A	26.7 µm	120.1 µm
PMN-PT 1-3 composite [103]	0.5 mm × 0.5 mm	34 MHz	72%	N/A	92 µm	135 µm

**Table 6:** A summary of IVUS imaging performance of the reported dual frequency transducers.

Study	Transducer Configuration	Frequency		Aperture Size	Piezoelectric Material	Axial Resolution	Lateral Resolution	Penetration Depth
Qiu et al. [112]	Side-by-side	Low	36 MHz	0.7 mm × 0.7 mm	PMN-PT	78 µm	132 µm	N/A
		High	78 MHz	0.35 mm × 0.35 mm	PMN-PT	34 µm	106 µm	N/A
Yoon et al. [113]	Side-by-side	Low	48 MHz	0.57 mm × 0.57 mm	PMN-PT	27 µm	122 µm	N/A
		High	152 MHz	0.57 mm × 0.57 mm	LiNbO_3_	14 µm	40 µm	N/A
Lee et al. [114]	Side-by-side Oblong shaped focused	Low	35 MHz	0.5 mm × 0.5 mm	PZT-5H	40 µm	153 µm	N/A
		High	105 MHz	0.5 mm × 0.5 mm	LiNbO_3_	25 µm	46 µm	<1 mm
Lee et al. [115]	Side-by-side	Low	35 MHz	0.5 mm × 0.5 mm	PZT-5H	104 µm	180 µm	N/A
		High	70 MHz	0.5 mm × 0.5 mm	PZT-5H	28 µm	65 µm	N/A
Ma et al. [74]	Back-to-back	Low	35 MHz	0.5 mm × 0.5 mm	PMN-PT	46.0 µm	231.5 µm	5 mm
		High	150 MHz	0.5 mm × 0.5 mm	LiNbO_3_	17.2 µm	87.3 µm	0.5 mm
Munding et al. [111]	Back-to-back	Low	30 MHz	0.5 mm × 0.5 mm	PZT-5H	50 µm	224 µm	>5 mm
		High	80 MHz	0.27 mm × 0.27 mm	PZT-5H	16 µm	120 µm	<3 mm
Su et al. [116]	Back-to-back	Low	35 MHz	0.4 mm × 0.6 mm	PZT-5H	37 µm	199 µm	4 mm
		High	80 MHz	0.3 mm × 0.4 mm	PZT-5H	19 µm	128 µm	0.95 mm

**Table 7:** A comparison of IVUS harmonic image resolution with that of fundamental images.

Study	Transducer Configuration	Frequency	Aperture Size		Piezoelectric Material	Image Types	Axial Resolution	Lateral Resolution
Lee et al. [110]	Dual frequency Three elements formed a spherical shape	Low	35 MHz	0.5 mm × 0.5 mm	PZT-5H	Fundamental	75.5 µm	330 µm
		High	70 MHz	0.5 mm × 0.5 mm	PZT-5H	Fundamental	68.1 µm	110 µm
						Second harmonic	31.1 µm	70 µm
Lee et al. [114]	Dual frequency Dual elements formed a spherical shape	Low	35 MHz	0.5 mm × 0.5 mm	PZT-5H	Fundamental	40 µm	153 µm
		High	105 MHz	0.5 mm × 0.5 mm	LiNbO_3_	Fundamental	25 µm	46 µm
						Third harmonic	25 µm	46 µm
Lee et al. [115]	Dual frequency Dual elements spherically deformed	Low	30 MHz	0.5 mm × 0.5 mm	PZT-5H	Fundamental	70 µm	215 µm
		High	70 MHz	0.5 mm × 0.5 mm	PZT-5H	Fundamental	30 µm	112 µm
						Second harmonic	32 µm	155 µm
Ma et al. [125]	Dual frequency Dual element stacked vertically	Low	6.5 MHz	0.6 mm × 3 mm	PMN-PT	Superharmonic	35 µm	N/A
		High	30 MHz	0.6 mm × 0.5 mm	PMN-PT			
Martin et al. [134]	Dual frequency Dual element stacked vertically	Low	5.5 MHz	0.6 mm × 3 mm	PMN-PT	Superharmonic	N/A	N/A
		High	37 MHz	0.6 mm × 0.5 mm	PMN-PT			
Li et al. [138]	Dual frequency Dual element stacked vertically	Low	6 MHz	0.6 mm × 3 mm	PMN-PT	Superharmonic	N/A	N/A
		High	35 MHz	0.6 mm × 0.5 mm	PMN-PT			
Wang et al. [139]	Dual frequency Dual element stacked vertically	Low	2.25 MHz	0.37 mm × 5 mm	PMN-PT	Superharmonic	40 µm	N/A
		High	30 MHz	0.37 mm × 0.6 mm	PMN-PT			

**Table 8:** Comparison of different intravascular imaging modalities for the assessment of vulnerable plaque.

Imaging Modality	Fibrous Cap Thickness (<65 µm)	Lipid Pool Composition	Dimension Assessment	Inflammatory Reaction
IVUS	Poor	Necrotic core, microcalcifications, positive arterial remodeling	Excellent	Poor
OCT	Excellent	Microcalcifications, neo-angiogenesis, fibrous cup disruption, erosion and thrombus	Moderate	Excellent
NIRS	Moderate	Necrotic core	Poor	Not applicable
IVPA	Poor	Necrotic core	Poor	Moderate
NIRF	Poor	Necrotic core	Poor	Excellent
TRFS (FLIM)	Moderate	Necrotic core	Poor	Moderate

**Table 9:** A summary of different combined intravascular imaging modalities for the assessment of vulnerable plaque.

Imaging Modality	Characteristics of Vulnerable Plaques						Current Status
	Lumen Size	Plaque Burden	Lipid Pool	Fibrous Cap Thickness	Neo-Angiogenesis	Inflammation	
IVUS-OCT	Excellent	Excellent	Moderate	Excellent	Moderate	Poor	Commercially available
IVUS-NIRS	Excellent	Excellent	Excellent	Moderate	Not applicable	Not applicable	Commercially available
IVUS-IVPA	Excellent	Excellent	Moderate	Poor	Poor	Moderate	In vivo validation
IVUS-NIRF	Excellent	Excellent	Poor	Poor	Not applicable	Excellent	In vivo validation
IVUS-TRFS (FLIM)	Excellent	Excellent	Moderate	Excellent	Not applicable	Moderate	In vivo validation
IVUS-OCT-NIRF	Excellent	Excellent	Moderate	Excellent	Moderate	Excellent	Under development
IVUS-OCT-IVPA	Excellent	Excellent	Moderate	Excellent	Moderate	Moderate	Under development

**Table 10:** A summary of IVUS-based multi-modality intravascular imaging system performance.

Imaging Modality	Catheter Size	Transducer-Probe Arrangement	Transducer-Probe Parameter	Image Resolution	Penetration Depth	Frame Rate
IVUS-OCT	3.3 Fr [185] (Conavi Medical)	Co-linear arrangement	IVUS: 40 MHz OCT: 1310 nm	N/A	N/A	100/s (hybrid use)
	3.2 Fr [185] (Terumo)	Sequential arrangement	IVUS: 40 MHz OCT: 1300 nm	IVUS: 200 µm OCT: 15 µm	N/A	100–160/s (hybrid use)
	2.7 Fr [186]	Back-to-back	IVUS: 45 MHz OCT: N/A	N/A	N/A	10/s
	3.6 Fr [187]	Side-by-side	IVUS: 40MHz OCT: 1310 nm	IVUS: 57 µm OCT: 8 µm	N/A	20/s
IVUS-NIRS	3.2 Fr [188]	IVUS transducer and NIRS optics at 180° apart	IVUS: 40 MHz NIRS: 800–2500 nm	IVUS: >100 µm	IVUS: 8 mm NIRS: ~5 mm	IVUS: 16/s NIRS: 160 spectra/s
IVUS-IVPA	3.6 Fr [189]	Parallel alignment	IVUS: 35 MHz IVPA: 532 nm	IVUS: 59 µm	IVUS: 5 mm	N/A
	3.6 Fr [189]	Parallel alignment	IVUS: 80 MHz IVPA: 532 nm	IVUS: 35 µm	IVUS: 4 mm	N/A
	2.7 Fr [190] (Core size)	Sequential arrangement	IVUS: 40 MHz IVPA: 1210 nm	IVUS: 100 µm IVPA: 100 µm	IVUS: 4.5 mm IVPA: 4.5 mm	5/s
	3 Fr [191] (Core size)	Sequential arrangement	IVUS: 45 MHz IVPA: 1725 nm	IVUS: 52 µm IVPA: 60 µm	N/A	1/s
	3 Fr [192] (Core size)	Parallel alignment	IVUS: 40 MHz IVPA: 1725 nm	IVPA: 81 µm	N/A	25/s
	7.5 Fr [193]	Parallel alignment	IVUS: 40 MHz IVPA: 532 nm	IVUS: 36.3 µm IVPA: 48.5 µm	N/A	5/s
IVUS-NIRF	4.2 Fr [194]	Side-by-side	IVUS: 45 MHz NIRF: 750 nm	N/A	IVUS: 4 mm NIRF: 2 mm	30/s
	4.2 Fr [195]	Side-by-side	IVUS: 45 MHz NIRF: 780 nm	N/A	N/A	10/s
IVUS-TRFS (FLIM)	7 Fr [161]	Side-by-side	IVUS: 40 MHz FLIM: 300 nm	N/A	N/A	IVUS: 30/s FLIM: 6.7/s
	5 Fr [162]	Side-by-side	IVUS: 40 MHz FLIM: 390–629 nm	FLIM: 160 µm	N/A	IVUS: 30/s FLIM: 40/s
	3.7 Fr [196]	Side-by-side	IVUS: 40 MHz FLIM: 355 nm	N/A	N/A	IVUS: 30/s FLIM: 150/s
IVUS-OCT-NIRF	3.6 Fr [197]	Side-by-side	IVUS: 45 MHz OCT: 1310 nm NIRF: 635 nm	IVUS: 40 µm OCT: 8 µm	N/A	10/s
	3.9 Fr [198]	Side-by-side	IVUS: 40 MHz OCT: 1310 nm NIRF: 785 nm	N/A	N/A	20/s
IVUS-OCT-IVPA	6 Fr [199]	Side-by-side	IVUS: 40 MHz OCT: 1310 nm IVPA: 1250–1600 nm	N/A	>5 mm	20/s

## Data Availability

Not applicable.
